# Relation Between Injury Severity and Fractures of the First and/or Second Ribs in Blunt Chest Trauma at a Community‐Based Hospital: A Retrospective Study

**DOI:** 10.1155/emmi/4297853

**Published:** 2026-07-27

**Authors:** Ashraf F. Hefny, Sara E. Aldhaheri, Fatima Aldhaheri, Fatima Alameri, Sherif A. Fathi, Nirmin A. Mansour

**Affiliations:** ^1^ Department of Surgery, College of Medicine and Health Sciences, UAE University, Al Ain, UAE, uaeu.ac.ae; ^2^ Department of Surgery, Al Ain Hospital, Al Ain, UAE, alain-hospital.ae; ^3^ Faculty of Medicine, October 6 University, Cairo, Egypt, o6u.edu.eg; ^4^ Department of Family Medicine, SEHA Clinics, Al Ain, UAE

**Keywords:** blunt trauma, chest, community, fracture, hospital, rib, severity

## Abstract

**Objective:**

Trauma is a major global health concern that contributes significantly to morbidity and mortality worldwide. Road traffic collisions (RTCs) are the most common cause of blunt chest trauma (BCT). First and/or second rib fractures (FSRFs) are often indicative of high‐energy trauma. This study investigated the incidence, mechanisms of injury, management, and outcomes of FSRF in patients with BCT at a community‐based hospital.

**Methods:**

Data were retrospectively collected from all patients with BCT admitted to Al Ain Hospital between December 2014 and January 2020. Data for patients with FSRF were specifically reviewed. Information was obtained from the Al Ain Hospital Trauma Registry, which included all patients of all age groups who were admitted to the hospital or died in the emergency department during the study period.

**Results:**

During the study period, 958 patients with BCT were admitted to our institution, of whom 86 (9%) had FSRF. The most common mechanism of trauma was RTC in 62 (72.1%) patients. Patients with FSRF had significantly higher injury severity scores than those of patients with BCT without FSRF. Three patients with FSRF died (overall mortality, 3.5%) compared with 17 (1.9%) patients without FSRF. This difference was not statistically significant (*p* = 0.341, chi‐squared test).

**Conclusions:**

This study is consistent with previous reports demonstrating that FSRFs are associated with high‐energy trauma and potentially serious intrathoracic and extrathoracic injuries. The importance of these fractures in blunt trauma should be recognized to endorse close monitoring and timely therapeutic intervention, which is supported by existing literature.

## 1. Introduction

Trauma remains a major global health concern, contributing significantly to morbidity and mortality worldwide [1]. Among the various causes of blunt chest trauma (BCT), road traffic collisions (RTCs) are the most prevalent [2, 3]. The clinical presentation of BCT ranges from minor soft‐tissue injuries to severe, life‐threatening conditions, such as flail chest, cardiac injury, and major vascular disruption [4].

First and/or second rib fractures (FSRFs) are uncommon among patients with blunt trauma. These ribs are well protected by strong bony and muscular structures, including the sternum and clavicle anteriorly and scapula posteriorly [5]. Consequently, fractures of these ribs typically require a substantial force to the thoracic wall [5, 6]. Anatomically, these ribs lie near critical neurovascular structures within the thoracic outlet, including the subclavian artery and vein, brachial plexus, and sympathetic trunk. Accordingly, such fractures are often indicative of high‐energy trauma and are frequently associated with major thoracic and extrathoracic injuries [7].

Despite the recognized anatomical and clinical importance of upper rib fractures, their role as predictors of overall trauma severity and outcomes remains underexplored, particularly within community hospital settings in the Gulf Cooperation Council countries of the Middle East. The rapid economic expansion of the United Arab Emirates (UAE), similar to other high‐income countries in the Middle East, has led to a substantial increase in expatriate workers engaged mainly in high‐risk occupations such as construction and manual labor. This demographic distribution influences both the mechanisms and patterns of injury, with non‐UAE nationals being disproportionately exposed to occupational and high‐energy trauma. Therefore, this study aimed to investigate the incidence, mechanisms of injury, management, and outcomes of FSRF in patients with BCT at a community‐based hospital.

## 2. Patients and Methods

Data from all patients with BCT admitted to Al Ain Hospital between December 2014 and January 2020 were retrospectively collected. Information for patients with FSRF was specifically reviewed. Data were obtained from the Al Ain Hospital Trauma Registry, which included all patients of all age groups who were admitted to the hospital or died in the emergency department during the study period. Nonblunt mechanisms of injury were excluded from the study cohort. The trauma registry does not include patients who died at the scene or before reaching the hospital or those discharged from the emergency department. Any missing information was retrieved from patients’ electronic medical records. The collected data included age, sex, nationality, mechanism of injury, vital signs on admission, associated injuries, Glasgow Coma Scale (GCS) score, injury severity score (ISS), new ISS, abbreviated injury scale score, length of hospital stay, intensive care unit (ICU) admission, ICU length of stay, management, and outcomes.

The AIS coding was performed by a trained trauma registry nurse using the AIS 2005 version with the 2008 update. The ISS and NISS were automatically generated by the trauma registry based on the recorded AIS codes. To ensure data accuracy, AIS coding underwent regular quality assurance procedures, including random sampling audits of registry entries. These audits were conducted by specialist and consultant surgeons, with verification against clinical records (including radiological and operative reports) to confirm the correctness of injury coding and minimize data entry errors.

The severity of injuries and outcomes among patients with FSRF were compared with those among patients with BCT but without FSRF.

The primary outcome of this study is the incidence of FSRF among patients with BCT. The secondary outcomes included mechanisms of injury, associated injury patterns, management approaches (including operative and non‐operative interventions), and clinical outcomes such as the need for intubation, duration of mechanical ventilation, and length of hospital stay.

All statistical analyses were performed using IBM SPSS Statistics (Version 28). Data are reported as medians (IQR) or numbers (%). For continuous or ordinal data, the Mann–Whitney *U* test was used to compare two independent groups. Differences between categorical variables were tested using Pearson’s chi‐square test or Fisher’s exact test, as appropriate. Fisher’s exact test was applied when the assumptions of the chi‐square test were not satisfied. Statistical significance was defined as a *p* value < 0.05.

Informed consent was obtained from all the patients who were admitted to the hospital or from their guardians. This study was approved by the Tawam Human Research Ethics Committee of Abu Dhabi, UAE (T‐HREC Ref. MF2058‐2023‐952).

## 3. Results

During the study period, 958 patients with BCT were admitted to our institution, 86 (9%) of whom had FSRF. The basic patient characteristics are shown in Table 1.

**TABLE 1:** Baseline characteristics of patients with first and/or second rib fractures (*n* = 86).

Demographics and vital signs	
Male, *n* (%)	76 (88.4%)
Female, *n* (%)	10 (11.6%)
Age (years), median (IQR)	33 (24)
Systolic blood pressure (mmHg), median (IQR)	131 (38)
Pulse (beats/min), median (IQR)	90 (25)
Respiratory rate (breaths/min), median (IQR)	19 (3)
GCS score, median (IQR)	15 (0)
^∗^Common thoracic injuries *n* (%)	
Pneumothorax	46 (53.5%)
Pulmonary contusion	43 (50%)
Surgical emphysema	14 (16.3%)
Hemothorax	11 (12.8%)
Flail chest	7 (8.1%)
Sternum fractures	4 (4.7%)
Great vessel injury	1 (1.2%)
^∗^Extrathoracic injuries *n* (%)	
Head injury	36 (41.9%)
Upper extremity injury	33 (38.4%)
Spine injury	26 (30.2%)
Lower extremity injury	23 (26.7%)
Face injury	18 (20.9%)
Abdominal injury	17 (19.8%)
Neck injury	10 (11.6%)
Injury severity scores, median (IQR)	
ISS	13 (8)
NISS	17 (13)
Hospital length of stay (days), median (IQR)	4 (7)

Abbreviations: ISS, injury severity score; NISS, new injury severity score.

^∗^The percentage exceeds 100% because some patients sustained more than one injury.

The median (IQR) age of patients with BCT and FSRF was 33 (24) years; seven (8.1%) patients were younger than 18 years. Non‐UAE nationals were the most commonly admitted patients, comprising 71 (82.5%). A total of 83 patients (96.5%) sustained polytrauma. The most common mechanism of injury was RTC, observed in 62 patients (72.1%), followed by falls from a height greater than 1 m in 15 patients (17.4%). Of those involved in RTCs, 47 (75.8%) resulted from car‐to‐car collisions, of whom 30 (63.8%) were car drivers.

The most common thoracic and extrathoracic injuries are listed in Table 1. During their management, 10 (11.6%) patients received blood transfusions, and 13 (15.1%) patients underwent chest tube insertion. Nineteen (22%) patients were admitted to the ICU.

Pneumothorax, multiple rib fractures (more than one rib fracture), surgical emphysema, flail chest, and pulmonary contusion were significantly more frequent in patients who had FSRF than in other patients with BCT. In the overall cohort of patients with BCT, 72 patients (7.5%) sustained a single rib fracture, whereas 267 patients (27.8%) had multiple rib fractures. Injury severity, as measured by both the ISS and the NISS, was significantly higher in patients with multiple rib fractures compared to those with a single rib fracture (*p* = 0.007 and *p* = 0.002, respectively, Mann–Whitney *U* test). One patient with FSRF sustained a great vessel injury, compared with five patients without the FSRF group; however, this difference was not statistically significant (*p* = 0.434; Fisher’s exact test).

In the FSRF group, 41 patients sustained fractures of four or more ribs. Among them, 31 patients (75.6%) had associated lung contusions, which was significantly higher compared to patients with fewer than four rib fractures (*p* = 0.008; Pearson’s chi‐square test).

The incidence of upper limb injury was also significantly higher in patients with FSRF than in the other patients (Table 2).

**TABLE 2:** Thoracic and extrathoracic injuries in patients with BCT and first and/or second rib fractures (*n* = 86) compared with those in patients with BCT without these fractures (*n* = 872).

Injury	First and/or second rib fractures *n* (%)	Other patients with BCT *n* (%)	Total	*p* value^a^
Pulmonary contusion	43 (50)	284 (32.6)	327 (34.1)	**0.001**
Hemothorax	11 (12.8)	67 (7.7)	78 (8.1)	0.105
Pneumothorax	46 (53.5)	177 (20.3)	223 (23.3)	**< 0.001**
Multiple rib fractures	51 (59.3)	219 (25.1)	270 (28.2)	**< 0.001**
Surgical emphysema	14 (16.3)	40 (4.6)	54 (5.6)	**< 0.001**
Flail chest	7 (8.1)	9 (1)	16 (1.7)	**< 0.001**
Sternum fracture	4 (4.7)	37 (4.3)	41 (4.3)	0.869
Great vessel injury	1 (1.2)	5 (0.6)	6 (0.6)	0.513
Heart injury	1 (1.2)	10 (1.1)	11 (1.1)	0.995
Head	36 (41.9)	159 (38.9)	375 (39.1)	0.589
Face	18 (20.9)	339 (17.2)	177 (18.5)	0.539
Neck injury	10 (11.6)	86 (9.9)	96 (10)	0.603
Abdomen injury	17 (19.8)	215 (24.7)	232 (24.2)	0.313
Spine injury	26 (30.2)	255 (29.2)	281 (29.3)	0.848
Upper extremity	33 (38.4)	241 (27.6)	274 (28.6)	**0.036**
Lower extremity	23 (26.7)	231 (26.5)	254 (26.5)	0.959

*Note:* *p* < 0.05 considered significant. The bold values indicate statistically significant differences.

Abbreviation: BCT, blunt chest trauma.

^a^Pearson’s chi‐square test.

Patients with BCT and FSRF had significantly higher pulse rates, ISS, and new ISS than those of patients with BCT without FSRF. However, there was no significant difference in GCS scores between the two groups (Table 3).

**TABLE 3:** Vital signs and injury severity scores in patients with first and/or second rib fractures (*n* = 86) compared with those in other patients with BCT (*n* = 872).

Variable	Statistics	First and/or second rib fractures	Other patients with BCT	*p* value^a^
		(*n* = 86)	(*n* = 872)	
Pulse (bpm)	Median	90	86	**0.007**
	Minimum	61	0	
	Maximum	151	180	
	IQR	24	21	

Systolic BP (Hg mm)	Median	131	134	0.113
	Minimum	61	0	
	Maximum	199	218	
	IQR	37	24	

RR (per minute)	Median	19	18	0.104
	Minimum	11	0	
	Maximum	45	54	
	IQR	3	2	

GCS score	Median	15	15	0.444
	Minimum	3	3	
	Maximum	15	15	
	IQR	0	0	

ISS	Median	13	9	**< 0.001**
	Minimum	1	1	
	Maximum	75	75	
	IQR	8	10	

NISS	Median	17	9	**< 0.001**
	Minimum	1	1	
	Maximum	75	75	
	IQR	13	12	

*Note:* Statistical significance is set at *p* < 0.05. LOS, hospital length of stay. The bold values indicate statistically significant differences.

Abbreviations: BCT, blunt chest trauma; GCS, Glasgow Coma Scale; ISS, injury severity score; NISS, new injury severity score.

^a^Mann–Whitney test.

There were no significant differences between the two groups in demographics, mechanisms of injury, cardiac enzyme levels, or conservative management (non‐operative management for both thoracic and extra‐thoracic injuries). Nineteen (22.1%) patients with FSRF demonstrated elevated cardiac troponin enzyme levels. However, no echocardiographic features suggestive of cardiac contusion were identified in these patients. The admission to the ICU, chest tube insertion, and thoracic operations were significantly higher in patients with FSRF than in the other groups of patients with BCT (Table 4). Thoracic operations included thoracotomy and surgical fixation procedures such as open reduction and internal fixation of the sternum and ribs. Nonthoracic operations comprised procedures performed for associated injuries, including laparotomy, spinal fusion, and open reduction and internal fixation of upper or lower limb fractures.

**TABLE 4:** Demographics, mechanisms, cardiac enzymes, and management in patients with first and/or second rib fractures (*n* = 86) compared with observations in other patients with BCT (*n* = 872).

	First and/or second rib fractures (*n* = 86)	Other patients with BCT (*n* = 872)	Total (*n* = 958)	*p* value^a^
Demography				
Male sex	76 (88.4%)	752 (86.2%)	828 (86.4%)	0.581
Age less than 18 years	7 (8.1%)	90 (10.3%)	97 (10.1%)	0.522
Mechanism				
RTC car collisions	47 (54.7%)	413 (47.4)	460 (48%)	0.694
Car drivers	30 (34.9%)	312 (35.8)	342 (35.7%)	0.391
Work‐related injury	12 (13.9%)	126 (14.4%)	138 (14.4%)	0.945
Cardiac enzymes				
High troponin	19 (22.1%)	218 (25%)	237 (34.1%)	0.109
High creatine kinase	10 (11.6%)	72 (8.3%)	82 (8.6%)	0.302
High CK‐MB	10 (11.6%)	107 (12.3%)	117 (12.2%)	0.072
Management				
ICU admission	19 (22.1%)	67 (7.7%)	86 (8.9%)	**0.025**
Blood transfusion	10 (11.6%)	86 (9.9%)	96 (10%)	0.603
Chest tube insertion	13 (15.1%)	51 (5.8%)	64 (6.7%)	**0.001**
Conservative management	59 (68.6%)	631 (72.4%)	690 (72%)	0.439
Thoracic operations	8 (9.3%)	23 (2.6%)	31 (3.2%)	**0.005**

*Note:* *p* < 0.05 considered significant. The bold values indicate statistically significant differences.

Abbreviations: BCT, blunt chest trauma; CK‐MB, creatine kinase–myocardial band; ICU, intensive care unit; RTC, road traffic collision.

^a^Pearson’s chi‐square test.

Twelve (13.9%) patients with FSRF required intubation, which was significantly higher compared with 53 (6.1%) patients without FSRF (*p* = 0.011; Fisher’s exact test). The duration of intubation in the FSRF group ranged from 1 to 20 days, with a median of 7 days. Only one patient in this group developed ventilator‐associated pneumonia.

The ICU and hospital lengths of stay were significantly longer in patients with FSRF than in other patients with BCT (*p* = 0.035 and *p* = 0.005, Mann–Whitney *U* test, respectively).

In the current study, three patients with FSRF died (overall mortality, 3.5%) compared with 17 (1.9%) patients in the other group. However, the difference was not statistically significant (*p* = 0.341, chi‐squared test). Two of the patients who died had severe RTC, and the third fell from a height of 9 m. In all three patients, the cause of death was severe head injury.

## 4. Discussion

Although FSRFs are widely recognized as indicators of high‐energy mechanisms and potential markers of severe associated injuries [6], their incidence has not been well reported in general trauma databases. In our study, these fractures were present in 9% of all patients with BCT admitted to the hospital. This provides important data because the existing literature focuses on rib fractures collectively rather than on FSRF. Consistent with previous research, the clinical relevance of these fractures is linked to higher ISS, greater ICU admission rates, and increased risks of serious thoracic and extrathoracic injuries, reinforcing their role as markers of severe trauma [6, 7].

An important demographic observation in our study was that most patients with FSRF were non‐UAE nationals (83%), which aligns with the country’s population composition, in which expatriates represent most of the population. This demographic pattern is similar to that of other Gulf Cooperation Council countries owing to the predominance of expatriates working in infrastructure‐related occupations [8, 9]. Understanding the demographic context is essential for designing effective preventive intervention strategies [10].

In agreement with the existing literature, most of our patients (72%) sustained injuries because of RTC, followed by falls from heights [2, 6, 11]. Among patients with FSRF who were involved in car‐to‐car collisions, most of the injured patients (63.8%) were car drivers. Chest injuries in car drivers usually result from abrupt deceleration and thoracic wall compression against the steering wheel [12]. These injuries may be exacerbated if the patient remains unrestrained.

Abbas et al. showed low driver compliance with seatbelt laws in our community and, more broadly, across GCC countries in the Middle East [13]. Seatbelts are designed to protect the body by stabilizing and decelerating it during a crash, thereby decreasing the probability of occupant ejection from the vehicle or chest injuries caused by contact with interior vehicle structures [14, 15]. Public education and law enforcement aimed at improving seatbelt compliance among drivers and other car occupants may reduce the incidence of severe chest injuries [16, 17].

Rib fractures are comparatively less prevalent in the pediatric population than in adults, primarily because of the increased compliance and elasticity of the pediatric thoracic cage, which allows for deformation and force transmission without fracture [18]. Nevertheless, the present study did not demonstrate a statistically significant difference in the incidence of FSRF between adult and pediatric patients, a finding that may be attributable to the limited number of patients, which could have reduced the statistical power to detect differences between the groups.

In line with existing research, our findings revealed a significantly high incidence of serious thoracic injuries, such as flail chest, pneumothorax, and pulmonary contusions, among patients with FSRF. Such injuries are known to be associated with impaired pulmonary function, underscoring the need for close medical supervision and proactive patient management, especially in patients with flail chest associated with rib fractures [4, 12, 19, 20].

The study showed that injury severity was higher in patients with multiple rib fractures. In addition, patients with FSRF who sustained fractures of four or more ribs had significantly higher pulmonary contusion compared with patients with fewer than four rib fractures, which is in agreement with previously reported literature [21].

The study showed that there was no statistical difference in great vessel injury between patients with FSRF compared to patients without FSRF. It is important to acknowledge that fatal great vessel injuries, such as aortic rupture, are frequently associated with prehospital mortality [4]. Our trauma registry does not include patients who died at the scene, and therefore such cases could not be captured in this analysis. In addition, some patients who were dead on arrival at the hospital may also have sustained great vessel injuries. However, due to the absence of postmortem examinations, it was not possible to confirm the exact cause of death or the presence of associated vascular injuries in these cases.

This study demonstrated a significant correlation between upper extremity injuries and FSRF, likely caused by the anatomical connection of the ribs to the clavicle and scapula. These structures collectively participate in load transmission during BCT, making the upper extremity more susceptible to injury during severe blunt trauma [22, 23].

There were no significant differences between patients with FSRF and other groups of patients with BCT in terms of demographics, mechanisms of injury, or cardiac enzyme levels. While myocardial injury markers were elevated, there was no confirmatory imaging evidence of clinically significant cardiac contusion. However, chest tube insertion, thoracic operations, length of hospital stay, and intubation were significantly high in patients with FSRF. These findings are consistent with other studies showing that these fractures are often accompanied by critical injuries that require more thoracic surgery and prolonged management [12, 24–28].

The current study showed no statistically significant difference in mortality rates between patients with FSRF and other patients. However, the limited number of death events reduces the ability to detect a true association, and the study is likely underpowered to identify meaningful differences in mortality.

In another study, Sammy et al. investigated outcomes in a cohort of 1683 patients with first rib fractures compared to 8369 patients with fractures of other ribs and found that, although first‐rib fractures were associated with increased injury severity, the risk‐adjusted mortality was comparable between the groups. These findings suggest that, while certain rib fracture patterns may indicate more severe trauma, they do not necessarily translate into higher mortality after adjustment for confounding factors [29].

A lower GCS score is strongly associated with a high risk of mortality in polytraumatized patients; however, the GCS score reflects the severity of traumatic brain injury rather than overall injury severity across different body regions [12, 30]. In the current study, although there was no significant difference between patients with FSRF and other groups with regard to GCS scores, it is noteworthy that all three fatalities in the FSRF group were attributed to severe traumatic brain injuries. This finding is consistent with previous studies showing that traumatic brain injury is the most important factor in determining mortality in patients with polytrauma [31].

### 4.1. Limitations

This study was a retrospective analysis conducted at a single center with a relatively limited number of patients, which may limit multivariable analysis‐related outcomes. The retrospective design inherently constrained the availability of critical data elements that were not systematically recorded in the trauma registry or medical records, such as seatbelt usage. The severity of injury and mortality may also have been underestimated, as patients who died at the scene or those discharged directly from the emergency department without hospital admission were not captured in the registry. In addition, due to the observational nature of the study and the fact that most patients sustained polytrauma, causal relationships cannot be established. And it is not possible to attribute severe injuries and outcomes solely to FSRF. This should be considered when interpreting the study findings.

Nevertheless, the findings of our study, which originated from a community‐based hospital, align closely with those reported by major trauma centers, thereby reinforcing the external validity and real‐world relevance of our results.

## 5. Conclusions

This study is consistent with previous reports demonstrating that FSRFs are associated with high‐energy trauma and potentially serious intrathoracic and extrathoracic injuries. The importance of these fractures in blunt trauma should be recognized to endorse close monitoring and timely therapeutic intervention, which is supported by existing literature. Most injuries resulting from RTCs occurred among drivers; therefore, preventive measures should focus on proper seatbelt use, enforcement of traffic safety laws, and public education on road safety. The findings of our study, which originated from a community‐based hospital, align closely with those reported by major trauma centers, thereby enhancing the external validity and practical applicability of our conclusions.

## Author Contributions

All authors contributed ideas for the research and editing.

Ashraf F. Hefny performed data collection, data analyses, and wrote the article; Sara E. Aldhaheri conceived and designed the study and reviewed and edited the manuscript; Fatima Aldhaheri contributed to study conception and design; Fatima Alameri contributed to study conception, writing, reviewing, and editing of the manuscript; Sherif A. Fathi contributed to study conception, writing, reviewing, and editing of the manuscript; and Nirmin A. Mansour contributed to study conception, writing, reviewing, and editing of the manuscript.

## Funding

No funding was received for the conduct of this study.

## Disclosure

This study was presented as a poster presentation in the 50th World Congress of the International Society of Surgery (ISS/SIC), Kuala Lumpur, Malaysia, in August 2024. All authors provided approval of the final manuscript.

## Ethics Statement

This study was approved by the Tawam Human Research Ethics Committee of Abu Dhabi, UAE (T‐HREC Ref. MF2058‐2023‐952).

## Conflicts of Interest

The authors declare no conflicts of interest.

## Supporting Information

Additional supporting information can be found online in the Supporting Information section.

## Supporting information


**Supporting Information** STROBE checklist cohort completed.

## Data Availability

The data that support the findings of this study are available from the corresponding author upon reasonable request.
