# Supplementary material for: Relation Between Injury Severity and Fractures of the First and/or Second Ribs in Blunt Chest Trauma at a Community‐Based Hospital: A Retrospective Study
Source: Emerg Med Int. 2026 Jul 27;2026:4297853. doi: 10.1155/emmi/4297853 (PMC13408435; doi:10.1155/emmi/4297853)
Supplement: Supplementary file 1 — Supporting Information STROBE checklist cohort completed. [file EMMI-2026-4297853-s001.docx]

**STROBE Statement Checklist – Cohort Studies**

Manuscript: Relation between injury severity and fractures of the first and/or second ribs in blunt chest trauma at a community-based hospital: A retrospective study

| Item | STROBE recommendation | Page(s) | Assessment |
| --- | --- | --- | --- |
| 1(a) | Indicate the study's design with a commonly used term in the title or the abstract | 1-2 | The title and abstract clearly state that this is a retrospective observational study of blunt chest trauma patients |
| 1(b) | Provide an informative and balanced summary | 2 | Yes, in the abstract section |
| 2 | Explain scientific background and rationale | 3 | The introduction outlines the clinical importance of upper rib fractures and their association with trauma severity. |
| 3 | State specific objectives and hypotheses | 3 | The study aims to evaluate the relationship between fractures of the first and/or second ribs in blunt chest trauma and injury severity and outcomes. |
| 4 | Present key elements of study design early | 4 | Retrospective observational cohort study design is clearly stated. |
| 5 | Describe setting, locations, dates | 4 | Study conducted in a community-based hospital (UAE) over the period between December 2014 and January 2020 |
| 6(a) | Eligibility criteria and selection of participants | 4 | (a) Included all patients with blunt chest trauma; exclusion criteria non blunt trauma. Patients identified from trauma registry/database. |
| 6(b) | Matching criteria (if matched study) | NA | Not applicable |
| 7 | Clearly define outcomes, exposures, predictors, confounders | 4 | Exposure: first/second rib fractures in blunt chest trauma; outcomes: ISS, ICU admission, intubation, mortality. |
| 8 | Data sources and measurement | 4 | Injury severity calculated using AIS 2005 update 2008 and ISS/NISS derived from trauma registry |
| 9 | Describe efforts to address bias | 11 | Potential biases (retrospective design, missing data) discussed in limitations. |
| 10 | Explain study size | 5 | Reported; no sample size calculation (acceptable retrospective registry). |
| 11 | Explain handling of quantitative variables | 4 | Variables categorized (e.g., first and/or second ribs fractures, ISS, NISS). |
| 12(a) | Describe statistical methods | 4 | Statistical comparisons between groups performed using appropriate tests (e.g., chi-square, t-test) |
| 12(b) | Methods for subgroups/interactions | 5-7 | Yes |
| 12(c) | Address missing data | 4 | Missing; mention retrieval from EMR but not missing-data handling. |
| 12(d) | Loss to follow-up | NA | Not applicable |
| 12(e) | Sensitivity analyses | NA | Not reported |
| 13(a) | Report participant numbers at each stage | 6 | Total included and analyzed patients clearly specified |
| 13(b) | Reasons for non-participation | 6 | Total included and analyzed patients clearly specified. |
| 13(c) | Consider flow diagram | NA | Not applicable |
| 14(a) | Participant characteristics | 19 | Yes (Table 1) |
| 14(b) | Missing data for each variable | NA | Not reported |
| 14(c) | Summarise follow-up time | NA | Not applicable |
| 15 | Report outcome events | 5-7 | Outcomes including ICU admission, intubation, and mortality reported |
| 16(a) | Main results with estimates and precision | 5-7 | Yes, Association between fractures of the first and/or second ribs in blunt chest trauma and injury severity reported with statistical significance |
| 16(b) | Category boundaries | NA | Not applicable |
| 16(c) | Translate relative risk to absolute risk | NA | Not applicable |
| 17 | Report other analyses | 6-7 | Comparative analyses between groups presented |
| 18 | Summarise key results | 12 | Yes |
| 19 | Discuss limitations | 11 | Yes, Limitations include retrospective design, single center, and small number of mortality events. |
| 20 | Interpretation | 7-11 | Yes, Results interpreted cautiously and aligned with previous literature. |
| 21 | Generalisability | 11 | Yes, Findings discussed in context of similar trauma populations |
| 22 | Funding and role of funders | 13 | No external funding / Funding source stated. |
